# High AGR2 protein is a feature of low grade endometrial cancer cells

**DOI:** 10.18632/oncotarget.25838

**Published:** 2018-07-31

**Authors:** Areege Kamal, Anthony Valentijn, Roger Barraclough, Philip Rudland, Nihad Rahmatalla, Pierre Martin-Hirsch, Helen Stringfellow, Shandya B. Decruze, Dharani K. Hapangama

**Affiliations:** ^1^ Department of Women's and Children's Health, Institute of Translational Medicine, University of Liverpool, Liverpool, UK; ^2^ The National Center for Early Detection of Cancer, Oncology Teaching Hospital, Baghdad Medical City, Baghdad, Iraq; ^3^ Institute of Integrative Biology, University of Liverpool, Liverpool, UK; ^4^ Lancashire Teaching Hospital NHS Trust, Preston, UK; ^5^ Liverpool Women’s Hospital NHS Foundation Trust, Liverpool, UK

**Keywords:** endometrial cancer, AGR2, metastasis, hormone regulation

## Abstract

**Background:**

Biomarkers for identification of endometrial cancers (ECs) with high risk of recurrence are required to reduce the rising EC-related mortality. AGR2 is a prognostic marker in several hormonally-regulated cancers.

**Aim:**

To assess the utility of AGR2 as a prognostic marker in EC.

**Methods:**

*AGR2* immunoexpression was evaluated in 163 human endometrial samples. Change in *AGR2* mRNA levels in response to oestrogen and dihydrotestosterone was studied *in vitro*.

**Results:**

Upregulation of AGR2 (protein and mRNA) was seen in low grade EC, compared to the postmenopausal endometrium (*P* = 0.013) and to the high-grade EC (*P* < 0.0001). Elevated AGR2 protein expression-scores were associated with a high expression of estrogen alpha (ERα), progesterone, androgen receptors and early clinical stages. Metastatic lesions maintained higher AGR2 expression relative to matched-primary tumors. High-AGR2 protein levels were associated with better overall survival (*P* = 0.02) in all ECs, but in highly-ERα-expressing ECs, AGR2 associated with unfavourable patient outcome. Androgen through its receptor, downregulated *AGR2* mRNA in the Ishikawa cells.

**Conclusions:**

AGR2 is overexpressed in low grade ECs and positively associated with hormone receptors. The association between high AGR2 and progressive disease within the high-ERα-expressing ECs suggests that in this group of patients, AGR2 might be a potential biomarker of poor prognosis.

## INTRODUCTION

Endometrial cancer (EC) is the most common gynecological cancer in the Western world and the incidence is expected to double by 2025 [1]; EC-associated mortality also is increasing in an era of decreasing cancer-related mortality in many other cancers [2]. Although hysterectomy is curative in most women with ECs, 2–15% of early stage and over 50% of advance stage ECs will eventually recur with poor patient outcome [3, 4]. The currently-available clinicopathological classification of EC patients into low, intermediate and high risk sub-groups has been criticized to be of limited specificity in stratifying patients for postsurgical management [5]. The recently proposed molecular classification of EC is projected to improve this, particularly after integrating the suggested 4 molecular groups with the existing clinicopathological subgroupings [6]; however, reliable bio-markers to predict response to therapy or recurrence during follow-up, are still lacking.

AGR2 is the human homologue of *Xenopus laevis* Anterior Gradient [7], a protein that belongs to the protein disulfide isomerase family (PDI) of endoplasmic reticulum-resident proteins [8]. PDI proteins fulfil roles associated with correct protein folding, maturation and secretion. AGR3 is a homologue of AGR2, sharing 71% sequence homology to AGR2 and was initially identified in breast cancer cell lines [9] and both found to be associated with estrogen receptor (ER) positive breast tumours [10]. AGR2 has been reported to be a prognostic marker in several hormonally-regulated cancers such as those of breast [11, 12], prostate [13] and ovary [14], where it is involved in drug resistance [15, 16] and metastatic growth [17]. AGR2 can also be secreted [18] and is detected in extracellular fluids [14, 19–21]; hence AGR2 protein has been proposed as a compelling biomarker for cancer detection and/or follow-up. Recently, higher AGR2 expression was found in ECs that developed in women previously treated with tamoxifen compared to those who had not been exposed to tamoxifen [22]. While many ECs are hormone responsive [23], the pattern of AGR2 expression and its possible role in endometrial carcinogenesis remains to be described.

In this study, changes in the levels of endometrial AGR2 protein/*AGR2* mRNA expression are shown for the first time across the normal pre and postmenopausal endometrium, premalignant atypical endometrial hyperplasia, EC and in matched metastatic lesions. The prognostic significance of AGR2 expression is also assessed in our cohort and further validated using The Cancer Genome Atlas (TCGA) EC series. Furthermore, we examined the regulation of *AGR2* gene expression by steroid hormones in the hormone-responsive Ishikawa cell line, an *in vitro* model of early EC.

## RESULTS

### Demographics

As expected, in our cohort, the premenopausal women were significantly younger than those in the postmenopausal (*P* < 0.0001) and EC (*P* < 0.0001) groups (Table 1). Although 10/12 (83%) of hyperplastic lesions were in the background of EC, they were significantly younger than other EC patients (*P* = 0.024). This was most pronounced compared to HGEC (*P* = 0.001). Within EC patients, all participants were postmenopausal; and women with HGEC were significantly older than those with LGEC (*P* = 0.02). Statistical analyses were corrected for multiple testing.

**Table 1:** Study groups and demographics

Study groups	No	%	Age^*^ (years)	BMI^*^ kg/m^2^
Proliferative phase	16		39 (30–49)	26.7 (18–46)
Postmenopausal	15		64 (52–79)	26 (22–38)
Endometrial hyperplasia with atypia	11		55 (48–73)	30.10 (24–57)
Endometrial cancer	100		67 (41–89)	30.7 (20–54)
LGEC	50	50	64 (41–84)	30.8 (22–54)
Grade1	30	30	64 (51–84)	32 (22–53)
Grade2	20	20	64 (41–77)	29 (22–54)
HGEC	50	50	70 (51–89)	29.6 (20–43)
Grade3	15	17	69 (51–83)	26.7 (22–43)
Serous	8	8	73 (64–82)	29.8 (25–35)
Clear cell	12	12	69 (52–82)	29.9 (25–32)
Carcinosarcoma	15	15	78 (59–89)	24.2 (20–37)
Metastatic lesions	16		68 (41–89)	–

^*^Data expressed as median (range).

### AGR2 expression in human endometrium

**Figure 1:**
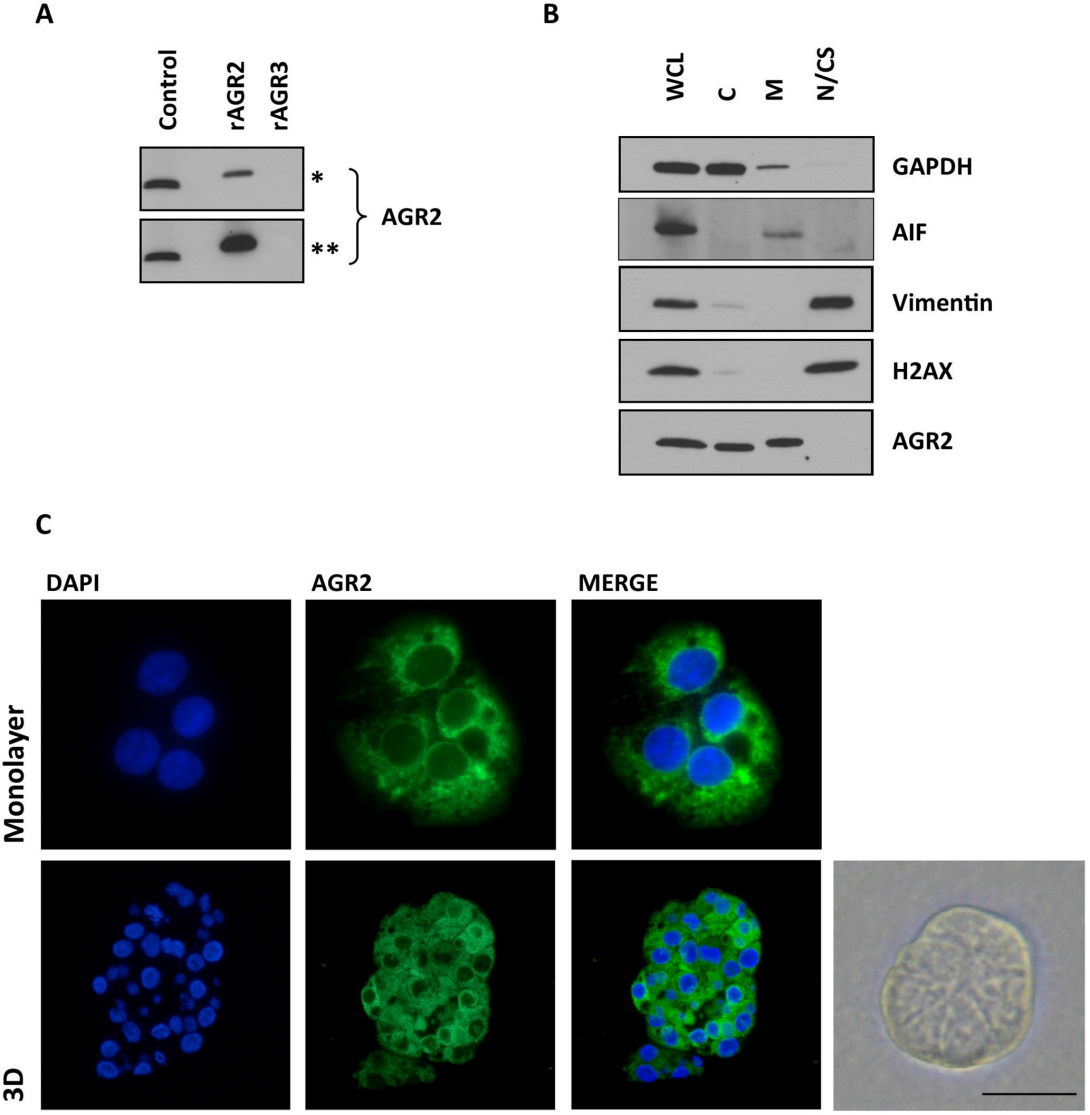
The pattern of AGR2 staining and specificity (**A**) The specificity of Rabbit monoclonal anti-human AGR2 antibody to recombinant AGR2 (rAGR2) and AGR3 (rAGR3). 10 ng of each recombinant protein was loaded per lane. Control, 25 mg total protein from a cell lysate from MEF280 cells. Asterisks denote short (^*^, 1 min) and long (^**^, 5 mins) exposures. (**B**) Ishikawa cells were sub-fractionated as described. Whole cell lysate (WCL), cytoplasmic proteins (C), integral membrane and organellular proteins (M) and nuclear and cytoskeletal proteins (N/CS). AIF, apoptosis-inducing factor, is associated with the mitochondria. Subcellular fractionation is representative of three experiments. (**C**) AGR2 immunofluorescence of Ishikawa cells, in both monolayer and 3D culture. The bright field image is a representative image of Ishikawa cells forming a spheroid 3D culture. Scale bar 50 μm.

**Figure 2:**
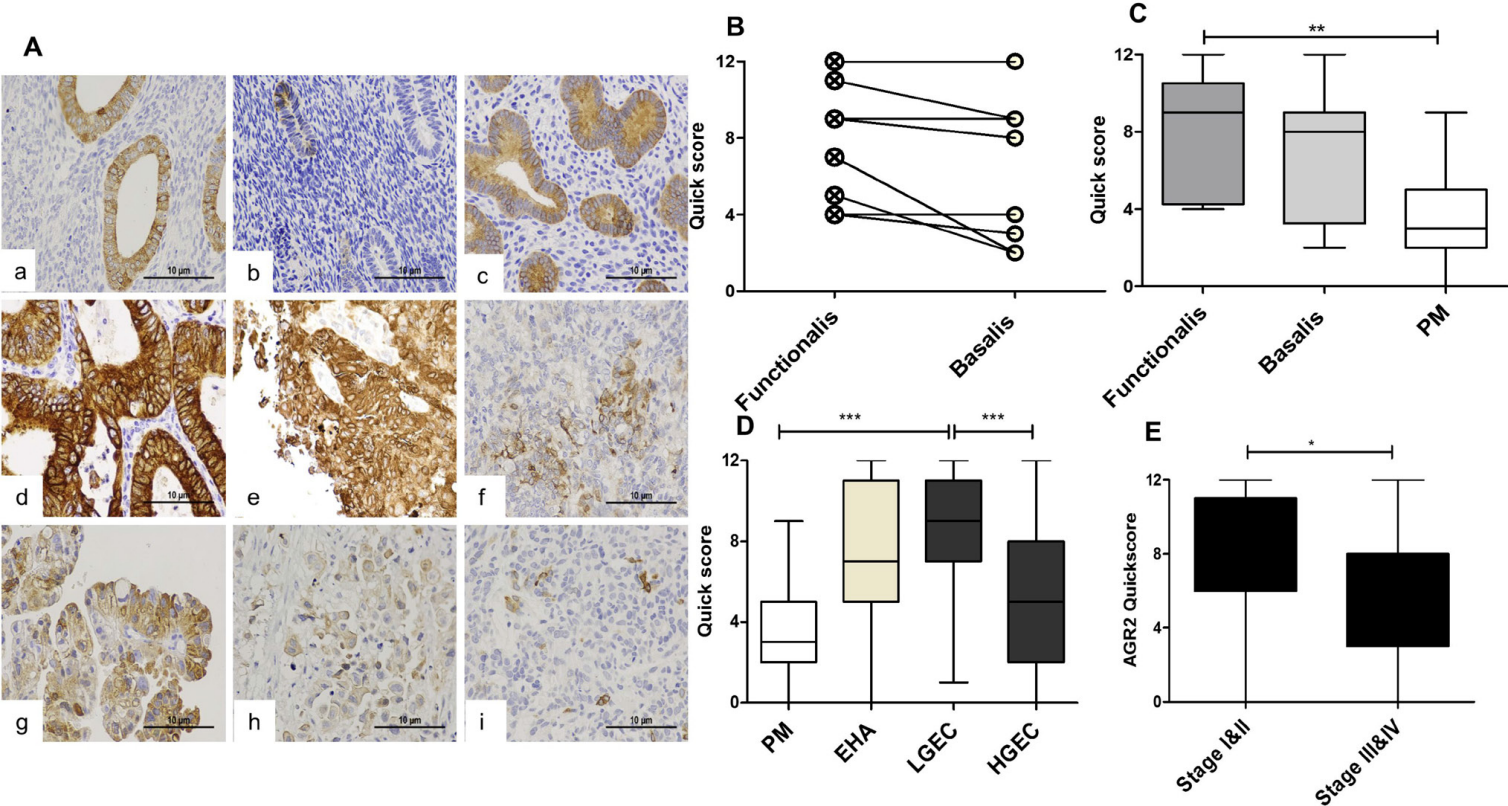
The immunoexpression of AGR2 in in normal and premalignant and malignant human endometrium (**A**) Microphotographs illustrate the expression of AGR2 in the cytoplasm of normal proliferative phase (a), and postmenopausal endometrium (b); hyperplastic endometrium (c); endometrioid endometrial cancer grade1–3 (d–f), serous (g), clear cell (h) and carcinosarcoma (i) positive stain appears brown. (Abcam Catalog# 2574-1, rabbit monoclonal, 1:1500, magnification ×400). Immunoscores of AGR2 in (**B**) Proliferative phase functionalis vs basalis layers (*n* = 12). (**C**) Proliferative phase layers vs postmenopausal endometrium, PM (*n* = 15). (**D**) PM; endometrial hyperplasia with cytological atypia, EHA (*n* = 11); histopathologically classified low grade cancer, LGEC (*n* = 50); high grade cancer, HGEC (*n* = 50). (**E**) Early clinical stages I and II (*n* = 60) vs advance clinical stages III and IV (*n* = 36).

Since AGR2 is a highly related homologue to AGR3, sharing 71% sequence homology, we initially sought to determine whether the antibody to AGR2 cross-reacted with AGR3. The antibody only reacted with recombinant AGR2 and not with recombinant AGR3 as assessed by immunoblotting (Figure 1A). Initially, the pattern of staining and specificity of the AGR2 antibody was characterized in the Ishikawa cells, grown in both monolayer and 3D culture. Immunofluorescence staining localized AGR2 protein to the cytosol in a diffuse and punctate pattern (Figure 1C). Subsequent subcellular fractionation of Ishikawa cells confirmed that AGR2 was associated with the cytosolic and membrane fractions; without apparent nuclear localization (Figure 1B). AGR2 protein was observed by immunohistochemistry in the epithelial cells of normal, pre- and post-menopausal endometrium and in the neoplastic human endometrium. In all cases, the stromal compartment did not show any AGR2-immunoreactivity (Figure 2A).

Functionalis layer of normal proliferative phase endometrium expresses higher AGR2 than the basilis layer

The immuno-expression of AGR2 in proliferative phase epithelium ranged between moderate to strong and the quick-scores were generally higher in the functionalis compared with the matched basalis (*P* = 0.021, Figure 2B). The basalis AGR2 quick-scores remained static across the menstrual cycle and in PM endometrium (not shown); however, these scores were significantly lower in the PM endometrium when compared with the proliferative phase functionalis (*P* = 0.004, Figure 2C) suggesting hormonal regulation.

Hyperplastic endometrial epithelium with cytological atypia (EHA) demonstrated significantly higher AGR2 quick-scores (*P* = 0.008) compared with the PM group (Figure 2D), but the observed statistical significance was lost when adjusted for multiple testing (*P* = 0.116).

### AGR2 is upregulated in low grade but not high grade ECs

**Figure 3:**
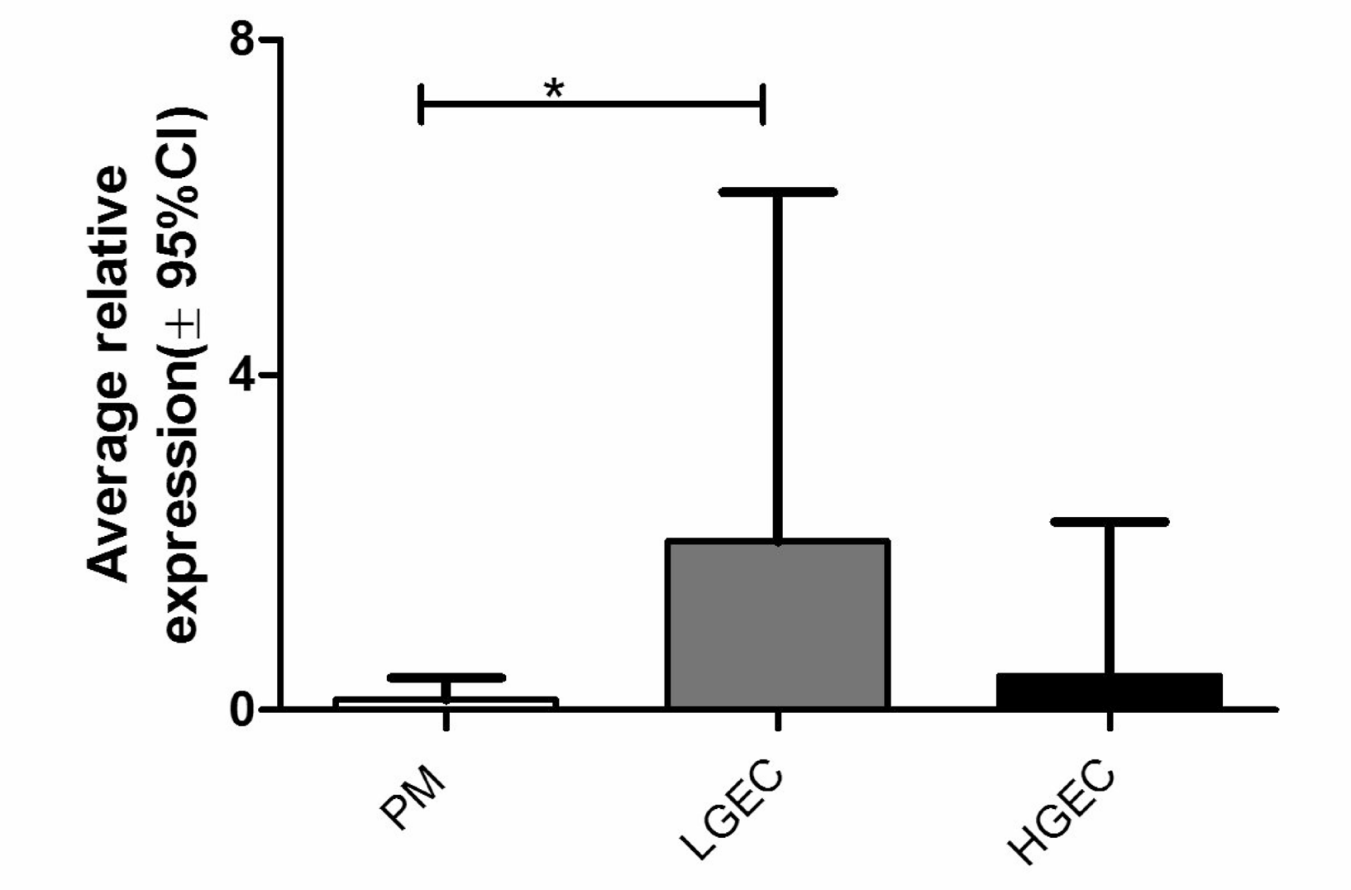
Average *AGR2* mRNA level in human endometrial samples relative to the geometric mean of WYHAZ and PPIA; PM, postmenopausal; LGEC, low grade; HGEC, high grade endometrial cancer

AGR2 protein was expressed in 92% of EC cases and in all (100%) LGEC. Compared with the healthy PM, AGR2 quick-scores were significantly higher in EC (Dunn’s test, *P* = 0.013) especially in LGEC (Dunn’s Test, *P* < 0.0001, Figure 2D). In contrast, the expression of AGR2 in HGEC was significantly less than in LGEC (*P* < 0.0001, Figure 2D), and was not statistically different compared with PM. Similarly, within endometrioid EC, grade 3 ECs showed a significant reduction in AGR2 quick-scores compared with grades 1 and 2 (Mann Whitney test, *P* = 0.016). Moreover, early clinical stage EC (I and II) showed higher AGR2 quick-scores than advanced stages III and IV (Mann Whitney test, *P* = 0.007, Figure 2E). The immunohistochemistry data was further supported by qRT-PCR study of the level of *AGR2* mRNA in human endometrial tissue (Figure 3). *AGR2* transcripts were detected at a higher level in LGEC compared with both PM (*P* = 0.015) and HGEC (*P* = 0.072).

### Metastatic lesions maintain AGR2

**Figure 4:**
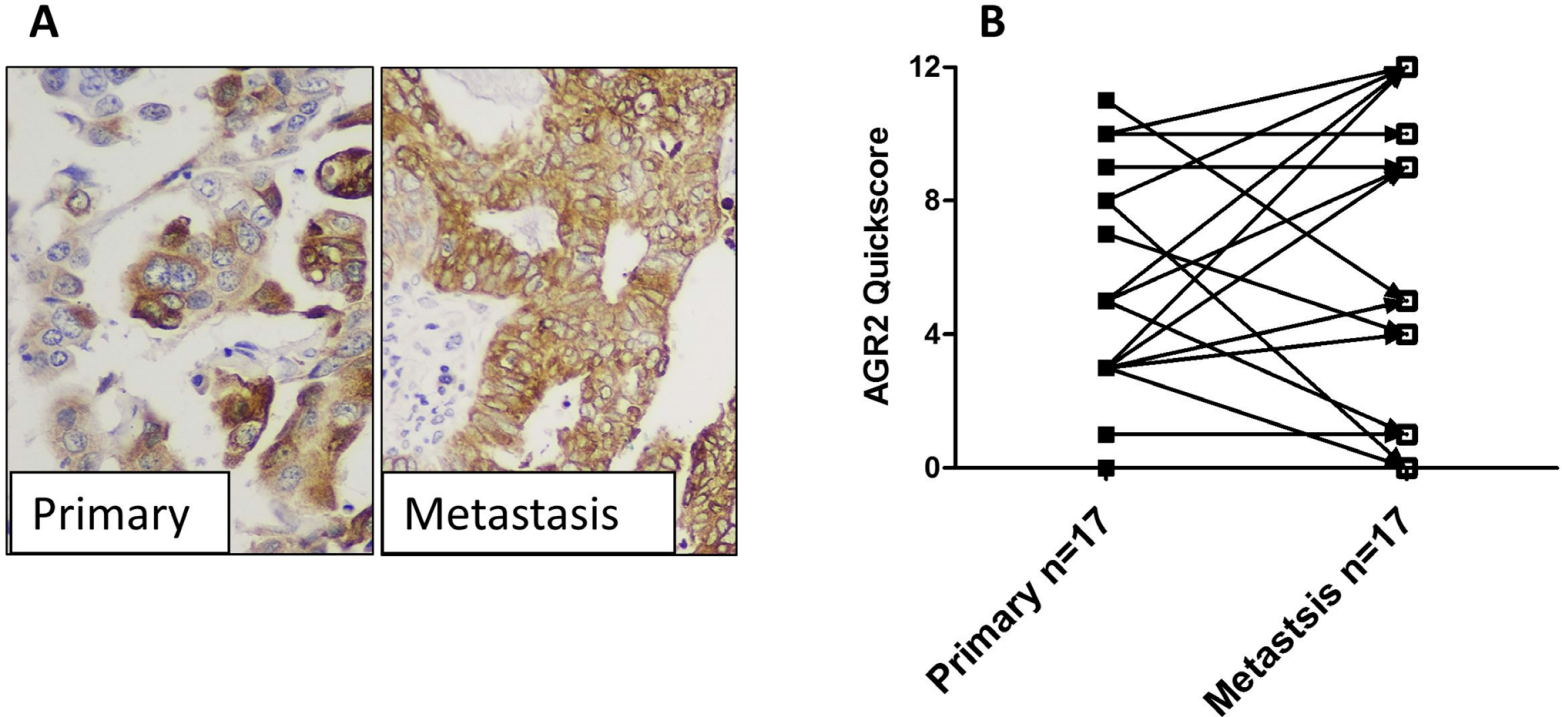
The expression of AGR2 in metastatic lesions (**A**) photomicrographs showing the immunoexpression of AGR2 in HGEC primary endometrial cancer and matched metastatic lesion (×400 magnification). (**B**) Immunoscores of AGR2 in primary tumours vs matched metastases.

AGR2 was expressed in most (14/17 (82%)) metastatic lesions and, although the proportion of cells expressing AGR2 protein, regardless the intensity, was significantly higher in metastatic lesions compared with the matched primary tumour cells (data not shown), the quick-scores (which collated immunostaining intensity and proportion of positive cells) were not significantly different between the 2 groups (Figure 4). This may suggest that most metastatic cells may undergo possible stress that increases the likelihood of individual cells expressing AGR2 but not necessarily increasing their intensity. Interestingly, metastatic lesions of both LGEC (2/2, 100%), and HGEC (10/15, 66%) demonstrated an apparent increase of AGR2 quick-scores when compared with their matched primary tumours as shown in Figure 4A and 4B.

The upregulation of AGR2 is associated with steroid hormone receptors expression in human EC

When the association of quick-scores for AGR2 expression of ≥5 with clinicopathological parameters was determined, no significant association was observed with AGR2 expression and the deep myometrial invasion, lymphovascular space invasion, cervical stromal invasion or extra-uterine invasion. However, ECs with AGR2 immunoscores of ≥5 were strongly significantly associated with LGEC (*P* < 0.0001) and stages I–II (*P* = 0.003, Table 2). In addition, high AGR2 immunoscores were significantly associated with EC specimens that were positive for androgen receptor (*P* = 0.038), progesterone receptor (*P* = 0.043), or estrogen receptor α (*P* = 0.010). This result is consistent with previous findings of the hormone responsiveness of AGR2, and more importantly, is consistent with regard to the general hormone receptor status of LGEC and HGEC, thereby providing an explanation of AGR2 being upregulated in LGEC compared with HGEC.

**Table 2:** The association of AGR2 expression with the clinicopatholgical parameters in endometrial cancer

AGR2							
Variables		Total	<5	(%)	≥5	(%)	*P*
Age	*≤*65	100	14	30	33	70	0.401
	>65		21	40	32	60	
BMI	<30	68	11	34	21	66	0.606
	≥30		10	28	26	72	
Grade	LG	100	8	16	43	84	<0.0001
	HG		27	55	22	48	
Stage	I–II	98	15	25	46	75	0.003
	III–IV		20	54	17	46	
Myometrial invasion	<50	97	16	28	41	72	0.085
	≥50		18	45	22	55	
Cervical invasion	–	97	23	33	47	67	0.485
	+		11	41	16	59	
LVI	–	98	17	42	23	58	0.2
	+		17	29	41	71	
LN	–	20	5	62	3	38	0.67
	+		6	50	6	50	
Extra-uterine invasion	–	96	14	45	17	55	0.168
	+		19	29	46	71	
AR	–	86	16	50	16	50	0.038
	+		15	28	39	72	
PR	–	86	20	47	23	53	0.043
	+		11	26	32	74	
ERα	–	86	8	73	3	27	0.01
	+		23	31	52	69	
ERβ	–	86	1	100	0	0	0.36
	+		30	35	55	65	
ERα/ERβ	Low	86	8	35	15	65	0.883
	high		23	37	40	63	

### Survival analysis

**Figure 5:**
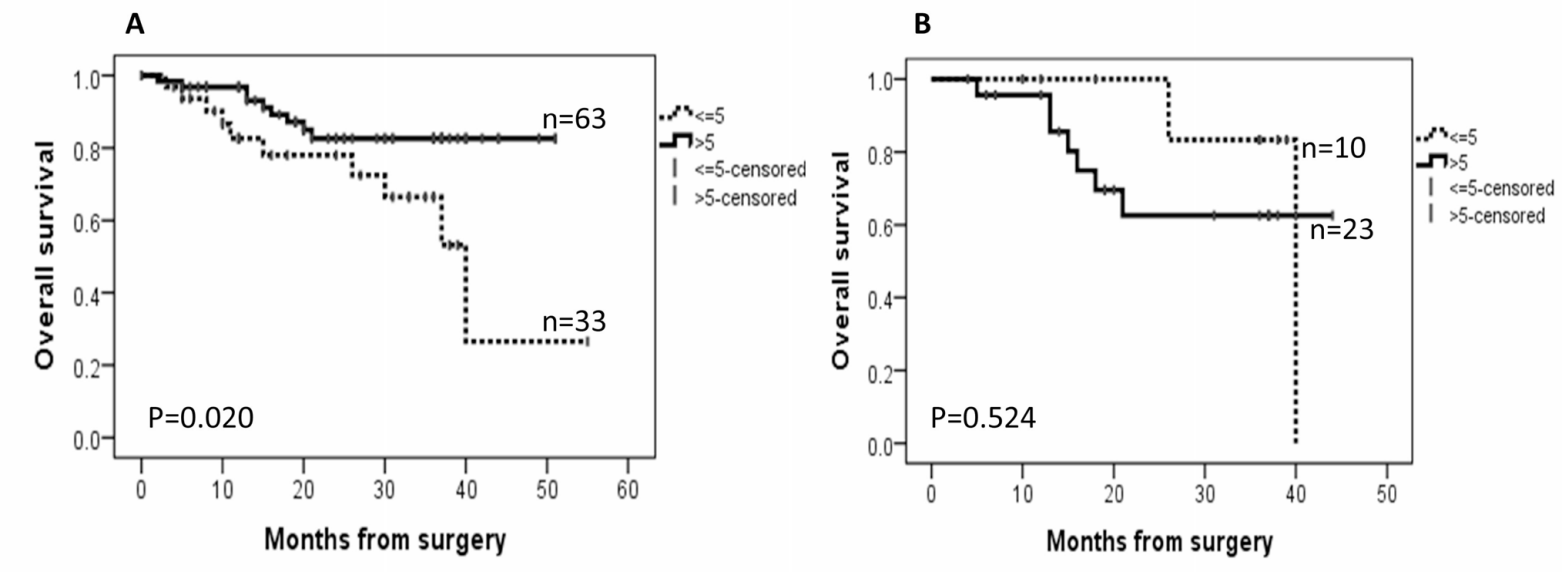
Kaplan–Meier survival curves for the correlation between the immunoexpression of AGR2 and overall survival (**A**) in endometrial cancer cohort; (**B**) in a subset of the cohort with oestrogen receptor (ER) immunoscores >6.

Follow-up data were available for all EC patients. By August 2017, the median follow-up was 19 months, ranging from 6 to 40 months. During the follow-up period, there were 5 recurrent tumours and 17 deaths (14 as a result of disease progression and 3 from other causes). AGR2 was associated with better overall survival of the EC patients when all cancer subtypes were analysed together (*P* = 0.020, Figure 5A), but no significant effect was seen on disease-free survival. For ECs with high ER immunoscores >6, high AGR2 appears to associate with shorter overall survival, however statistically that was not significant (*P* = 0.524, Figure 5B).

Validating our *in vivo* data on TCGA dataset and other published microarray data

Altered *AGR2* RNA expression levels with the tumour grade and stage, similar to our IHC data were also observed in the TCGA data (Supplementary Figure 2A). High *AGR2* RNA expression in the TCGA cohort was associated with longer overall survival (*n* = 589, clear separation of the KM plot, although *p* > 0.05, Supplementary Figure 2B). When only the high ERα-expressing group (*n* = 86) was considered, a similar trend to our IHC data was observed with high *AGR2* RNA associating with poor survival (Supplementary Figure 2C). However, the small sample size with very few recorded events precluded statistical testing of this apparent observation. Furthermore, there was a significant positive correlation between *AGR2* and *AGR3* RNA expression in the EC samples (*p* = 3.3E-48, *r* = 0.58, Supplementary Figure 3).

We have also validated our data by interrogating 3 further independent publically available EC microarray sample sets, to include 63 Grade 1 endometrioid cancers and 68 Grade 3 Endometrioid and Serous EC. Significant decrease in *AGR2* gene expression was noted with Grade 3 and Serous cancers compared with Grade 1 endometrioid cancers (Supplementary Figure 4).

Further *in silico* analysis highlighted *AGR2* gene expression to be perturbed by the mutation or knockdown of many drivers of endometrial carcinogenesis, such as *PTEN, KRAS* and *TP53*, (Supplementary Table 2) suggesting an increase in *AGR2* expression is an important feature of low grade ECs. Significantly high *AGR2* expression was observed *in PTEN, KRAS* (the commonest mutations seen in endometrioid cancers) mutated cancers compared with wild type cancers (*p* = 5.29E-8 and *p* = 4.74E-4 respectively, results not shown), and contrastingly low *AGR2* levels were seen in *TP53* mutated cancers (commonest mutations seen in high grade/ type 2 cancers) compared with the wild type cancers in the TCGA dataset (*p* = 9.98E-8, results not shown). This data further support the data generated from our study cohort, suggesting higher AGR2 is an important feature of low grade ECs.

### Androgen treatment downregulates the transcript for *AGR2*

In order to understand the relationships between *AGR2* expression and hormone receptor status, an *in vitro* culture model of Ishikawa (ISK) cells was used.

**Figure 6:**
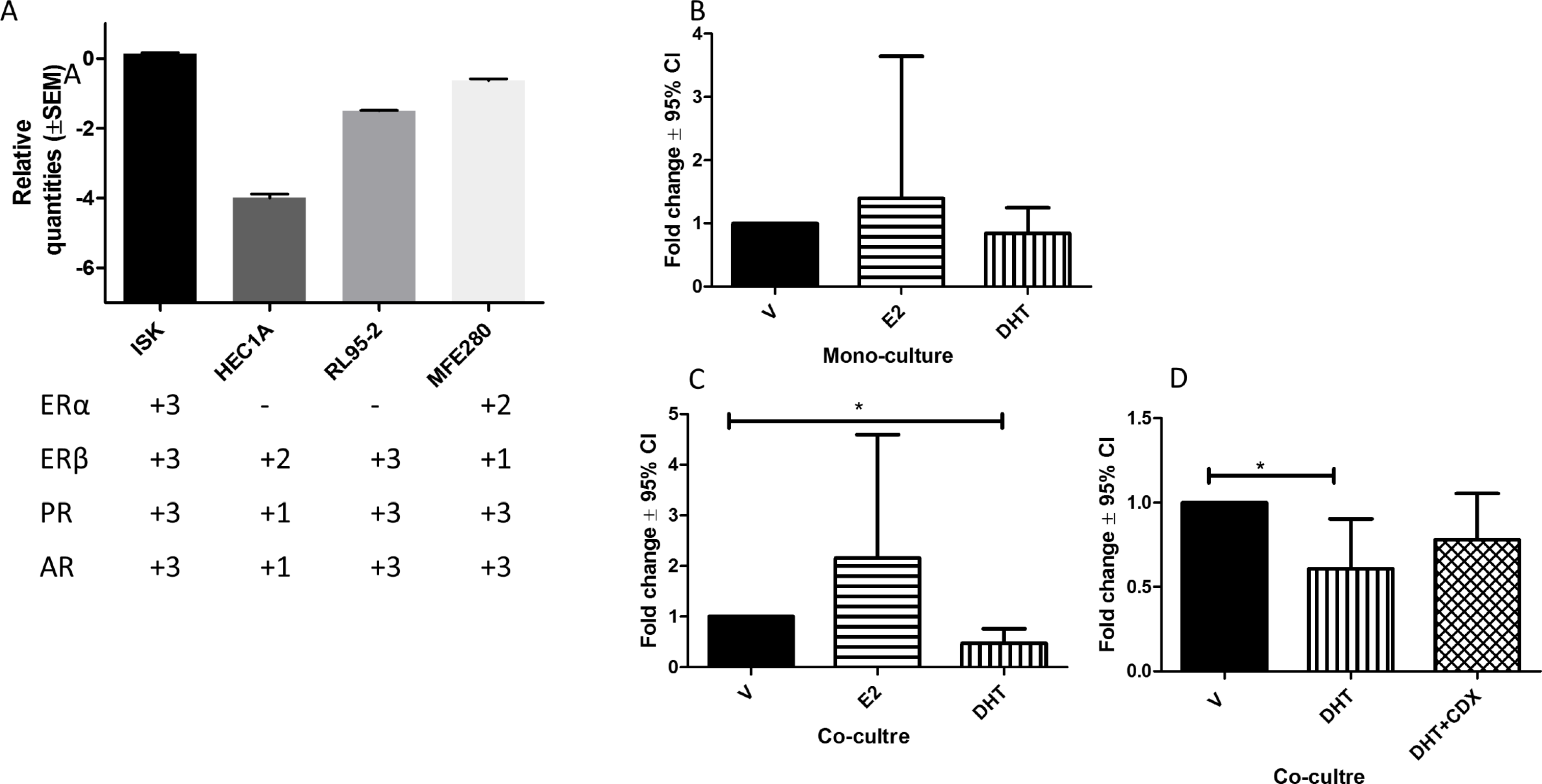
The expression of *AGR2* mRNA level using RT-qPCR (**A**) The bar graph represents the relative mRNA levels for *AGR2* in four different endometrial cancer cell lines and the plus score underneath represents the expression of steroid receptors mRNA for the corresponding cell line; (**B**) *AGR2* mRNA in Ishikawa cell after 24 h treatment with 1 × 10^–8^ M estradiol (E2) or 1 × 10^-6^ M dihydrotestosterone (DHT); (**C**) in Ishikawa cells co-cultured with hESC treated with E2 and DHT; D) in Ishikawa cells co-cultured with hESC 24 h treatment with DHT with and without casodex (CDX) pre-blocking. *N* = 6 for each experiment.

We initially characterized four EC cell lines for steroid hormone receptor [24] and *AGR2* expression (Figure 6A). All four EC cell lines expressed *AGR2* mRNA (Figure 6A). All 4 steroid receptors were expressed only in ISK cells that was established from a well-differentiated Grade-1 human endometrial adenocarcinoma [25] representing LGEC in our cohort, therefore, was chosen to ascertain the effect of estrogen and androgen on the *AGR2* transcription. There were no significant changes detected in the *AGR2* mRNA levels after E2 or DHT treatment in monocultures of ISK cells (Figure 6B). However, when they were cultured with hESC (co-culture system), the DHT treatment significantly reduced *AGR2* mRNA (*P* < 0.031), while it was not altered by E2 (Figure 6C). Maximum reduction of *AGR2* mRNA was seen after 24 h of DHT-treatment but the levels subsequently returned to the base line by 72 h (Supplementary Figure 1B). This DHT-effect in the co-culture system was partially reversed by AR antagonist (Figure 6D).

## DISCUSSION

We describe for the first time, the expression of AGR2, as assessed by IHC, in EC subtypes and the modulatory effect of steroid hormones on the transcript for *AGR2*, data supported by the TCGA.

Immunofluorescence and immunohistochemistry staining of Ishikawa cells and endometrium show an intracellular staining pattern for AGR2 that is in part consistent with localization to the endoplasmic reticulum. AGR2 has a KTEL motif in its C-terminal domain that directs it to the endoplasmic reticulum through binding to one of three KDEL receptors [26, 27]. Different affinities for the KDEL receptors may impact on the subcellular localization of AGR2 [28] such that AGR2 could be found in the cytoplasm, the endoplasmic reticulum and the extracellular environment [26]. In support of this, subcellular fractionation of Ishikawa cells showed that AGR2 was detectable in both the cytoplasmic and membrane fraction. However given the low affinity of KTEL for KDEL receptors, it is conceivable that the detergents used in the fractionation might have disrupted its association thereby promoting its exit from the endoplasmic reticulum and contributing to the cytoplasmic pool. Available data and *in silico* analyses demonstrated *AGR3* may co-express with *AGR2* or uncoupled in both human healthy tissues and carcinomas in stomach, colon, pancreas, breast, female reproductive system, or respiratory system in a tissue specific manner [29]. *AGR3* expression in the endometrium is not yet reported. We have confirmed the specificity of the antibody we used and thus the data we report is specific to AGR2.

Increased expression of AGR2 was observed in the premalignant EHA, with levels increasing further in LGEC compared with normal post-menopausal controls. AGR2 was not only significantly downregulated in type II EC as recently reported by others [22] but also in grade III endometrioid compared to grade I and II cancers. This suggests that increased AGR2 might be an early event in EC development. A larger sample size is always desired however, in addition to our substantial cohort, we interrogated data available in the larger TCGA cohort and in 3 other publically available, individual microarray dataset to justify our hypothesis. The collective agreement of data from all independent cohorts justify our hypothesis that high AGR2 is a feature of low grade ECs. Furthermore, many driver mutations in TCGA dataset were also associated with upregulation of *AGR2*. This high AGR2 appears to be a feature of low grade EC is seemingly at odds with its metastatic promoting property. Evidence exists from other tumours that cells with upregulated AGR2 acquire a metastatic, proliferative and invasive phenotype [17, 30]. Breast cancer cell lines transfected with AGR2 produced metastasis in a xenograft model [17], showed gain of anchorage-independent growth and promoted tumor growth [31]. Although a metastatic role for AGR2 in EC is yet to be demonstrated, overexpressing *AGR2* or added recombinant AGR2 in culture medium significantly increased proliferation in endometrial derived cancer cell lines [22]. Conversely, upon *AGR2* silencing, proliferation was reduced [22]. This would suggest a pro-proliferative role for AGR2 in EC. Such effects of AGR2 on proliferation have also been demonstrated in breast cancer cell lines [32, 33]. The positive expression of AGR2 in metastatic cytokeratin positive endometrial cells is speculated to be a consequence of several factors: These lesions also expressed high levels of AR and ERα [34]. Settlement of metastatic cells in the new microenvironment of the secondary tissue is a stressful event in cellular terms [35]. The expression of steroid receptors can add a further endocrine stimulated stress by inducing large alterations in gene transcription [21]. The involvement of AGR2 in protein folding and endoplasmic reticulum-assisted degradation [36] may, therefore, allow tumour cells to avoid cell death. Furthermore, a recent report suggests that AGR2 expression downregulate EMT process and maintains the epithelial phenotype [37] which metastatic cells may need in the new metastatic niche.

Previous studies from other hormone-responsive cancers, such as breast and prostate, reported oestrogen and androgen regulation of the *AGR2* gene [17, 20]. In the endometrium, the ovarian steroid receptor genes are well-established downstream targets of ligand (oestrogen) bound ERα [38]. In breast cancer studies, AGR2 expression is associated with ER-positive tumors and its overexpression is a predictor of poor prognosis. Moreover, the *AGR2* gene is directly targeted by ERα, which is preferentially bound in tumors with poor outcome [21]. We observed that the high AGR2 quick scores significantly correlated with positive protein expression scores for ERα, PR and AR, suggesting a possible role for E2 in AGR2 regulation. Since circulatory oestrogen and androgen hormones are significantly elevated in women with EC [39], we examined their effect on endometrial epithelial *AGR2* gene expression using two *in vitro* models: one monoculture; the other co-culture. In the absence of stromal cells, E2 and DHT failed to induce significant changes in *AGR2* mRNA levels in the Ishikawa cells. However this is in contrast to a recent study showing that both estrogen and tamoxifen induced AGR2 protein expression in Ishikawa cells after 24 hours [22]. Contrastingly, in our co-culture experiments, high dose DHT caused a modest, but significant reduction of *AGR2* mRNA. Although the AR antagonist did not completely restore the basal level of *AGR2* mRNA, we can conclude that down regulation of the *AGR2* gene can be, at least partially induced through AR. Physiological stress such as serum depletion and hypoxia, is seen as an alternative regulatory pathway for induction of AGR2 independent of steroid hormones [40].

## MATERIALS AND METHODS

### Study groups

The study was approved by Liverpool and Cambridge Adult Research Ethics Committee (LREC 09/H1005/55, 11/H1005/4, and CREC 10/H0308/75). As shown in Table 1, one hundred EC, 16 metastatic lesions (3 lymph node, 7 soft tissue, 3 parametrium, 3 omentum), 11 hyperplastic with cytological atypia EHA) and 31 full thickness normal endometrial biopsies were collected from patients undergoing hysterectomy in Liverpool Women’s Hospital and Lancashire Teaching Hospitals, Trusts from 2009–2014. The histological type and grade of EC specimens were assigned by experienced clinical gynecological pathologists in Lancaster and Liverpool as part of the routine clinical diagnostic workflow according to the International Federation of Gynecology and Obstetrics (FIGO) [19]. ECs were categorized as low grade (LGEC, including FIGO Grade 1 and Grade 2 endometrioid EC), or high grade tumours (HGEC, including FIGO Grade 3 endometrioid, serous, clear cell carcinoma and carcinosarcoma) [34, 41] for subsequent analysis of IHC data (Table 1). Proliferative phase specimens were assigned according to last menstrual date (LMP) and histological criteria [42]. All samples were divided into 2 parts; one fixed (≥24 h in 4% (v/v) buffered formalin) and paraffin-embedded for immunohistochemical staining and the other part immediately placed into RNA*later*^®^ (Sigma, Dorset, UK) for subsequent RNA extraction and qPCR.

Patient clinicopathological and demographic details were retrieved by review of hospital notes and clinical databases. Steroid receptors expression was available for 68 EC samples [34]. None of the patients received hormonal treatments, chemotherapy or pelvic radiation prior to surgery.

### Immunohistochemical staining and evaluation

Immunohistochemistry was performed as described previously [43, 44]. Briefly, 3 μm formalin- fixed, paraffin embedded, tissue sections were incubated with antibody specific to human AGR2 protein, steroid receptors, and to Ki67, all after antigen retrieval at pH6; antibody sources, concentrations, and incubation conditions are detailed in Supplementary Table 1. Primary antibody was detected using the ImmPRESS-polymer-based system (Vector Laboratories, Peterborough, UK) and visualizated with ImmPACT DAB (Vector Laboratories, Peterborough, UK) following manufacturer’s instructions, as previously described [43]. Sections were lightly counterstained in Gill’s Haematoxylin (Thermo Scientific, Runcorn, UK), dehydrated, cleared and mounted in synthetic resin. Matching isotype (0.5 µg/mL) replaced the primary antibody as a negative control, with internal positive control of a specific postmenopausal endoemtrial sample with known high AGR2 protein expression included in each staining run.

Levels of epithelial cytoplasmic AGR2 expression were semi-quantified using a quick score, consisting of a four-tiered scoring system for the % of epithelial cells stained positive for AGR2 protein (1%–10% = 1, 11%–30% = 2, 31%–50% = 3, and >50% = 4) combined with a score for the intensity of AGR2 staining (0 = no staining, 1 = weak staining, 2 = moderate staining and 3 = strong staining). The total score was produced by multiplying the score for the proportion of positive cells by the staining intensity category to achieve a final maximum total score of 12 per sample. Steroid receptors expression using Liverpool endometrial steroid quick score and proliferation index Ki67 were avaiable for 86 sample and were scored as described before [34].

ECs were then categorized according to AGR2 expression scores to compare the expression in relation to clinicopathological parameters. Several quick score cut-off points between 1 and 5 were tested to identify the AGR2 expression cutoff that distinguished the group with the worst clinicopathological features and worst outcomes (Supplementary Figure 1A). Out of the five tested cutoffs, a score of 5 showed the best categorization, thus a quick score of 5 was selected as the AGR2 expression cutoff score for this study, whereby <5 represent low AGR2 and scores ≥5 represent high AGR2.

### Immunofluorescence

Immunofluorescence was performed on 3µm paraffin embedded sections of Ishikawa spheroids, antigen retrieval at pH 6. Monolayers of Ishikawa cells grown in 8-well chamber slides (Sigma- Aldrich, Dorset, UK) were fixed in 10% NBF (Sigma- Aldrich, Dorset, UK) and permeabilised in 0.25% Triton X100 in PBS, 5 min at RT. Cells were stained with AGR2 antibody (1:100) with no primary antibody as control. Alexa Fluor 488 anti-rabbit IgG was from Cell Signalling Technology (Alexa FluorR 488, anti-rabbit). Nuclei were stained with DAPI in mounting medium (Vectashield, Vector Laboratories, Peterborough, UK). Immunofluorescence was visualised on a Nikon Eclipse 50i microscope using NIS-Elements F for image capture and Image J for processing.

### Real-time qPCR

Total RNA from tissue samples and from cell-line pellets was extracted using TRIzol Plus RNA Purification System (Life Technologies, Paisley, UK), and quantified by NanoDrop ND-1000 (Thermo Fisher Scientific, Loughborough, UK). Total RNA was reverse transcribed using AMV First Strand cDNA synthesis kit (New England Bio Labs, Hertfordshire, UK) after DNase treatment (DNase I (#M0303), New England Bio Labs, Hertfordshire, UK), using the manufacturer’s protocol as previously described [34]. cDNA was amplified by qPCR using JumpStart SYBR Green supermix (Sigma, Dorset, UK) and CFX Connect Real-Time System (Bio-Rad, Hertfordshire, UK) and the following primers: *AGR2* amplification were: forward 5′-ATTGGCAGAGCAGTTTGTCC-3′, reverse 5′-GAGCTGTATCTGCAGGTTCGT-3′ [45]; for Tyrosine 3-Monooxygenase/Tryptophan 5-Monooxygenase Activation Protein Zeta (YWHAZ), forward 5′-CGTTACTTGGCTGAGGTTGCC-3′, reverse 5′-GTATGCTTGTTGTGACTGATCGAC-3′ [46]; and for Peptidylprolyl Isomerase A (PPIA), forward 5′-AGACAAGGTCCCAAAGAC-3′ and reverse 5′-ACCACCCTGACACATAAA-3′ [47]. Relative transcript expression was calculated using the ΔΔCT method, normalised to the reference genes YWHAZ and PPIA using Biorad CFX manager.

### Cell culture

EC cell lines, Ishikawa, HEC1A, RL95-2, and MFE280 were purchased from commercial biobanks. The STR profile of the 4 obtained cell lines exhibited their published profile initially and at the end of our experimental process only with a few minor peaks indicating the beginnings of genetic drift but well within the 80–100% profile match as we previously described [24]. Immortalized normal human endometrial stromal cells, hTERT, were kindly donated by Dr Graciela Krikun of Yale University, USA [48]. Cells were maintained in 1:1 mixture of Dulbecco’s Modified Eagles’ Medium:Ham’s F-12 supplemented with 10% fetal bovine serum, 200 mM L-glutamine, and penicillin/streptomycin at 37° C in 5% CO_2_. RL95-2 cells required insulin supplementation in accordance with supplier instructions. For 3D culture of Ishikawa, 5000 cells were seeded in 50µl Matrigel (BD Biosciences) and cultured in the above medium for up to 14 days. All cell-culture reagents were obtained from Sigma-Aldrich (Dorset, UK) except where otherwise specified [24].

Since endometrial epithelial and stromal cell cross-talk is proposed to play a vital role in regulating endometrial growth and differentiation, ISK cells were co-cultured with immortalised human endometrial stromal cell line (hESC) to model the intimate interaction between endometrial epithelial and stromal cells *in vivo*. The cells were co-cultured using a Transwell system (0.4 μm pores; Corning): Ishikawa cells (3 × 10^5^ cells) in the insert (upper) and HESC (cell 1.5 × 10^5^) in the 6-well plate (bottom). Both cells lines were maintained in phenol red-free DMEM/F12 medium in the presence of 2% (v/v) charcoal-stripped FBS for 48 hours prior to hormone treatment. Steroid hormones, 0.01 μM 17 β-estradiol (E2; E8875-1G) and 1 μM 5α-dihydrotestosterone (DHT; D-073-1ML), individually or in combination with the AR antagonist, 1 μM Bicalutamide (Casodex (CDX); B9061-1 μg) were added and cells cultured for a further 24 hours. Stock solutions of hormones were made in methanol; control was methanol alone. All experiments were repeated six times with different batches of cultured cells.

### SDS-PAGE and immunoblotting

EC cell lines were extracted in RIPA buffer (50 mM Tris-Cl, pH8.0, 150 mM NaCl, 1% (v/v) NP-40, 0.5% (w/v) sodium deoxycholate and 0.1% (w/v) sodium dodecyl sulphate (SDS)) supplemented with protease (P8340, Sigma, Dorset, UK) and phosphatase inhibitors (PhosSTOP, Roche Diagnostics, West Sussex, UK). Lysates were analysed by SDS-PAGE under reducing conditions on precast 4–15% (w/v) gradient gels (Mini-PROTEAN TGX, Bio-Rad, Hertfordshire, UK) and transferred to polyvinylidene difluoride membrane (Bio-Rad). Recombinant AGR2 (ab151803) and AGR3 (ab152081) were from Abcam; 10 ng recombinant protein /lane. For subcellular analysis, Ishikawa cells were fractionated using a kit (#9038; New England Bio Labs, Hertfordshire, UK), which is based on differential detergent solubility and centrifugation, according to the manufacturer’s instructions. The following antibodies were used to assess the relative purity of each fraction: cytosolic, GAPDH (G8795, Sigma-Aldrich); membrane, AIF (Apoptosis-Inducing Factor, #5318, Cell Signalling Technology); cytoskeletal, vimentin (ab137321, Abcam) and nuclear, H2AX (ab 188819 Abcam). All used at 1:1000 apart from vimentin, 1:2000. HRP-linked secondary antibodies were from ThermoScientific, UK. Signal detection was performed using SuperSignalTM West Dura Chemiluminescent substrate (ThermoScientific) and CL-Xposure film (ThermoScientific).

### Statistical analysis

Statistical differences between groups were calculated by non-parametric tests (Kruskal–Wallis and/or Mann–Whitney *U*-test or Wilcoxon signed rank test) using Statistical Package for the Social Sciences (SPSS) version 21 and GraphPad Prism 5. Dunn’s test was used for small set multiple comparisons. Descriptive values were presented as median and range. The association between immuno-scores and the multiple clinicopathological parameters was examined with Pearson Chi-square tests. Disease-free survival and overall survival were calculated from the date of surgery to the date of recurrence/death or the date on which the patient was last seen. For survival analyses each parameter was categorized, and survival curves were obtained using the Kaplan Meier method. For all statistical tests, *P* < 0.05 was considered significant.

### TCGA Uterine cancer series

Experimental data were extended by examining *AGR2* RNA expression data obtained from the publicly-available The Cancer Genome Atlas (TCGA) cohort of uterine cancer with follow-up data using Illumina’s BaseSpace Cohort analyser application in BSCA [49] software; https://www.illumina.com/informatics/research/biological-data-interpretation/nextbio.html; Illumina, San Diego, CA, USA). TCGA contained RNA sequencing data with patient follow up data for *n* = 200 of LGEC (grade I and II endometrioid EC) and *n* = 281 of HGEC (grade III endometrioid, serous, undifferentiated EC). Published microarray datasets from 3 further individual studies were examined with Illumina’s BaseSpace Correlation Engine application in BSCA and genes that perturbed *AGR2* gene when mutated knocked down were identified with the Knockout atlas application as previously described [50] software;https://www.illumina.com/informatics/research/biological-data-interpretation/nextbio.html; Illumina, San Diego, CA, USA).

## CONCLUSIONS

Therefore, collectively, our data suggest, the upregulation of AGR2 is an early event in endometrial carcinogenesis and is associated with well differentiated tumours but may also contribute to the tumour progression and metastasis in a subset of those ECs. AGR2 expression in EC is likely to be under the regulation of ovarian hormones. The precise function of AGR2 and the mechanisms of its regulation in the normal and cancerous endometrium remain to be elucidated and warrant further investigation.

## SUPPLEMENTARY MATERIALS FIGURES AND TABLES




