# Supplementary material for: High AGR2 protein is a feature of low grade endometrial cancer cells
Source: Oncotarget. 2018 Jul 31;9(59):31459–72. doi: 10.18632/oncotarget.25838 (PMC6101135; doi:10.18632/oncotarget.25838)
Supplement: Supplementary file 1 [file oncotarget-09-31459-s001.pdf]

High AGR2 protein is a feature of low grade endometrial cancer cells

SUPPLEMENTARY MATERIALS

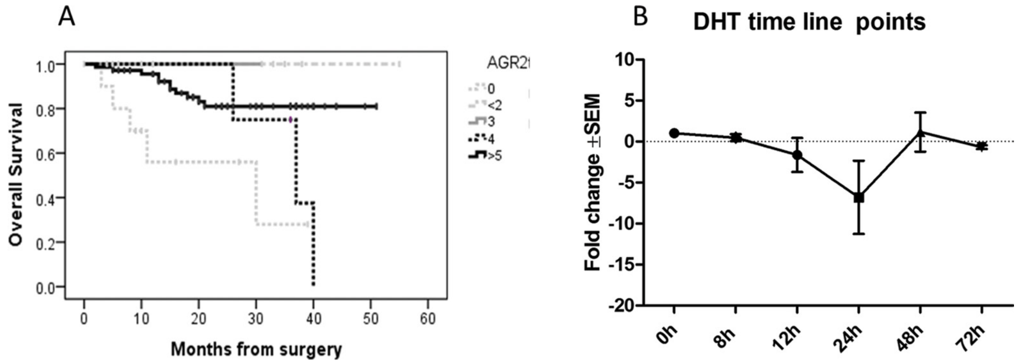

Supplementary Figure 1: (**A**) Kaplan–Meier curve with different AGR2 immunostaining quickscore cut off points; (**B**) *AGR2* mRNA level after treatment with 1 μM 5α-dihydrotestosterone (DHT; D-073-1ML) at different time points.

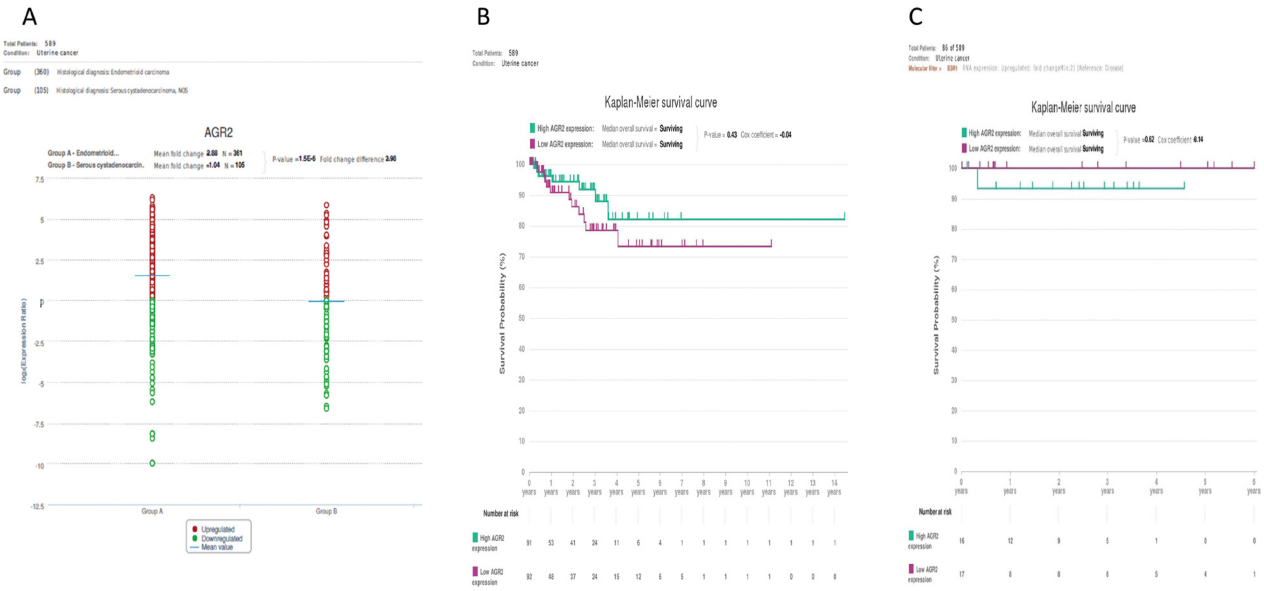

Supplementary Figure 2: Validations from TCGA Uterine cancer cohort data. (**A**) The expression of *AGR2* gene in Group A = endometrioid EC Group B = Serous EC. (**B**) Kaplan–Meier survival curves for the correlation between *AGR2* gene expression and overall survival in the entire endometrial cancer cohort (n = 589); (**C**) Kaplan-Meier survival curves for the correlation between *AGR2* gene expression and overall survival in EC patients with high *ERS1* (ER) RNA expression (n = 86). Data produced using Illumina's BaseSpace Cohort Analyzer application.

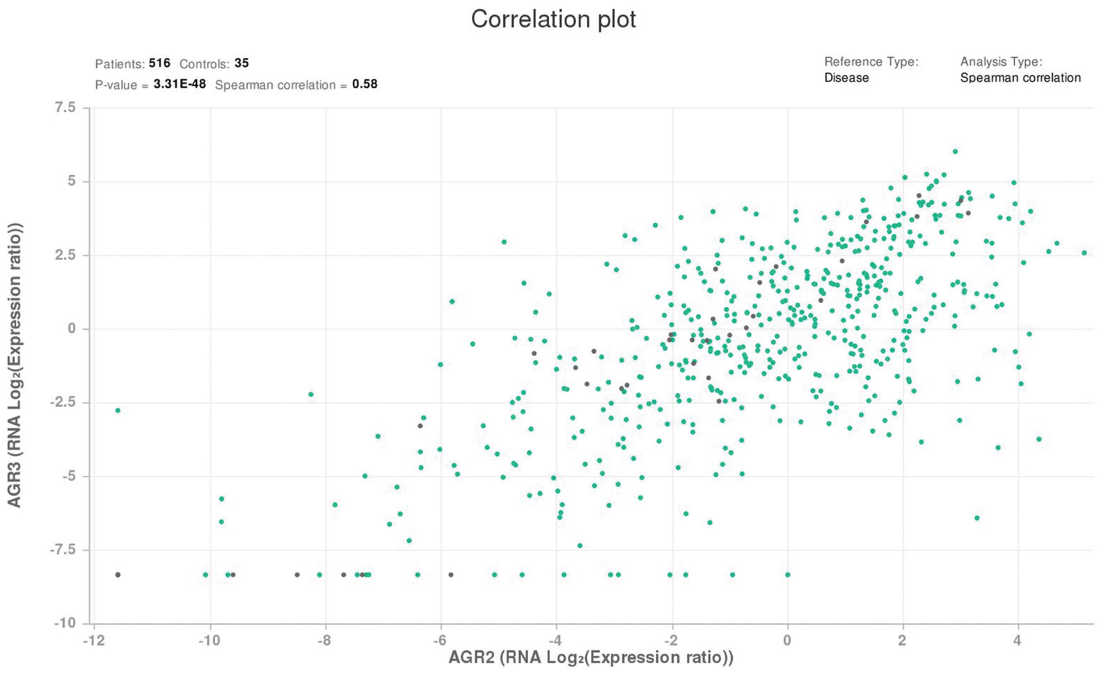

Supplementary Figure 3: Correlation of *AGR2* and *AGR3* RNA expression in TCGA cohort of uterine cancer samples. TCGA uterine cancer samples had RNA expression data for *AGR2* and *AGR3* in 516 endometrial cancer samples (green dots) and 35 control (black dots). The analysis demonstrated a significant positive correlation between the two genes (r = 0.58, p = 3.31E-48).

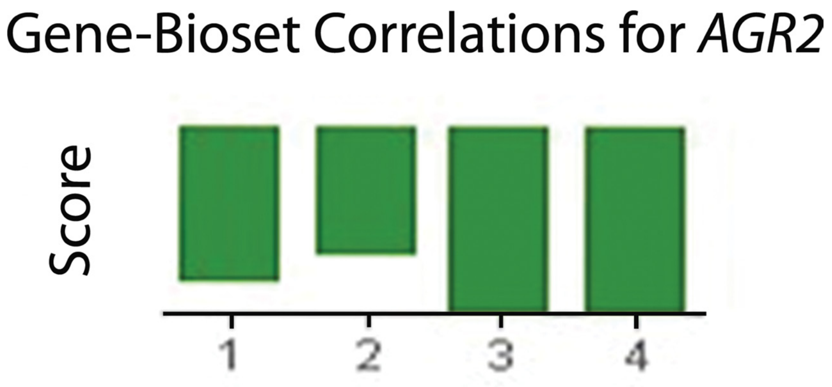

Supplementary Figure 4: External validation with 3 published EC microarray datasets. *AGR2* gene expression in 3 independent sample sets, using Illumina's BaseSpace Correlation Engine application (software; https://www.illumina.com/informatics/research/biological-data-interpretation/nextbio.html; Illumina, San Diego, CA, USA) to include 126 grade 1 endometrioid cancers, and 131 grade 3 endometrioid and serous EC from the following publically available microarray datasets; (1) GSE17025, 26 grade 3 versus 30 grade 1 endometrioid, (2); GSE24537, 11 serous versus 11 grade 1 (3); GSE2109, 31 grade 3 versus 22 grade 1 endometrioid, (4); GSE2109, 63 endometrioid grade 3 versus 22 endometrioid grade 1. The height of each vertical bar in the score matrix graph represents the score of the correlation between the analysed biosets and the *AGR2* gene. The correlation's score is based on the significance of the measurement made for the *AGR2* gene in the biosets analysed. The bar is green and appears below the midline depicting the *AGR2* gene was significantly down-regulated or deleted in in grade 3 and serous ECs compared with grade 1 ECs.

Supplementary Table 1: Primary antibodies and their immunohistochemistry conditions

Primary	Type	Clone	Supplier	HIAR*	Dilution	Incubation conditions	
Ab				(min)		Time (hour)	Temp (°C)
AGR2	Monoclonal	EPR3278	Abcam[2]	2	1:1500	20	4
AR	Monoclonal	441	DAKO[1]	2	1:50	20	4
PR	Monoclonal	PgR 636	DAKO	2	1:1000	1	18
ERα	Polyclonal		Abcam[2]	2	1:50	2	18
ERβ	Monoclonal	PPG5/10	Serotec[3]	2	1:50	20	4
Ki67	Monoclonal	MM1	Leica[4]	4	1:200	20	4

[*]Heat induced antigen retrieval by pressure cooking in citrate buffer pH 6 (Hapangama *et al*, 2012). [1]Ely, Cambridgeshire, UK; [2]Cambridge, UK; [3]Oxford, UK; [4]Newcastle upon Tyne, UK; Dorset, UK.

Supplementary Table 2: Genes that perturb *AGR2* when mutated or knocked down. The genes that influence *AGR2* expression when either mutated or knocked down were examined with Illumina's BaseSpace Knockout atlas application (software;https://www.illumina.com/informatics/research/biological-data-interpretation/nextbio.html; Illumina, San Diego, CA, USA). See Supplementary_Table_2
